# Response to the Letter regarding “Association between triglyceride-glucose (TyG) index and risk of depression in middle-aged and elderly Chinese adults: Evidence from a large national cohort study”

**DOI:** 10.17305/bb.2025.12760

**Published:** 2025-06-17

**Authors:** Zhang-Yang Xu, Yun-Xia Wang, Wen-Jun Su

**Affiliations:** 1 Department of Stress Medicine, Faculty of Psychology, Second Military Medical University, Shanghai, China; 2 Department of Nautical Psychology, Faculty of Psychology, Second Military Medical University, Shanghai, China

**Keywords:** Triglyceride-glucose index, TyG, insulin resistant, depressive symptom

## Abstract

This response addresses constructive feedback on our CHARLS cohort study linking the triglyceride-glucose (TyG) index to depressive symptoms. As noted by the letter’s author, a substantial body of research on TyG-related composite indices and mental disorders has emerged, with inconsistencies in cutoff values and the shape of dose-response curves observed among different indices. Our decision to focus solely on the single TyG index in this study was primarily motivated by the following two considerations. Firstly, the study population consisted of elderly individuals, among whom body mass index levels cannot effectively reflect metabolic status. Secondly, this study represents a continuation of our group’s preliminary preclinical research, thus specifically focusing on the factor of insulin resistance. Different findings derived from different indices necessitate consideration of various factors, including different application contexts, variations in the study populations, and differences in data processing methods. Finally, we believe more large randomized controlled trials and related pharmacological intervention studies are essential to validate the use of TyG and its related indices in the diagnosis and treatment of psychiatric disorders.

Dear Authors,

We sincerely appreciate your letter and your interest in our study. This research utilized the CHARLS database to design a prospective cohort study aimed at investigating the relationship between the insulin resistance index (TyG index) and the incidence of depressive symptoms. Our findings demonstrated a significant relationship between the triglyceride-glucose (TyG) index and the development of depression, with dose-response analysis revealing a J-shaped curve for this association after adjusting for relevant covariates [1].

After receiving your valuable letter, we carefully considered the issues you raised. Firstly, we noticed that the DOI and author names you cited for our article are incorrect. The correct citation is: Xu ZY, Zheng H, Pan ZJ, Hu SY, Wang YX, Su WJ. “Association between (TyG) index and risk of depression in middle-aged and elderly Chinese adults: Evidence from a large national cohort study.” Biomol Biomed. 2025;25(7):1621–30. doi: 10.17305/bb.2024.11800 [1]. Unfortunately, we were unable to locate the two references you mentioned: “TyG- body mass index (BMI) and depressive symptoms among middle-aged and older Chinese: an L-shaped association” and “Associations of TyG-related parameters and depressive symptoms with cardio-renal-metabolic multimorbidity in middle-aged and older adults.”

Nonetheless, the issues you raised are constructive and significant. Regarding the question of whether composite TyG indices (e.g., TyG-BMI, TyG-WC) are more effective than the standalone TyG index, we acknowledge the emerging research on TyG-derived composite indices in relation to mental disorders [2, 3]. It is undeniable that composite indices have certain advantages over single indices, particularly in their enhanced ability to reflect metabolic dysregulation. However, our study specifically selected the standalone TyG index to isolate the effect of insulin resistance as a singular factor. Notably, in older adults, BMI reflects not only fat mass but also muscle mass [4]. A low BMI could indicate frailty or sarcopenia, increasing the risk of depression, while a moderately elevated BMI might signify good nutritional status or sufficient muscle mass. Therefore, using BMI to obtain TyG-BMI may introduce a threshold effect at lower values, resulting in a differently shaped dose-response curve. In our multivariable regression models, we independently adjusted for BMI to isolate the independent contribution of insulin resistance to the risk of depression while holding BMI constant.

In our preclinical work, we have shown that mice subjected to chronic unpredictable mild stress (CUMS) exhibit co-occurrence of depressive-like behaviors and insulin resistance, and that treatment with the antidiabetic drug glyburide ameliorates both phenotypes by inhibiting NLRP3 inflammasome activation [5]. Based on these findings, we aimed to conduct a real-world study using a publicly available cohort to explore the relationship between insulin resistance and depressive symptoms.

As we know, different indices may exhibit varying predictive abilities for the same disease due to several factors. Firstly, the application scenarios differ; for instance, the thresholds used in large-scale health screenings differ from those employed in diagnostic settings. Secondly, the disease itself can exhibit heterogeneity; particularly in the case of psychiatric conditions, clinical presentations may vary across different stages. Lastly, differences in clinical study design, including variations in the baseline characteristics of enrolled participants, sample size, and data quality control (e.g., handling of missing data), may significantly impact the final conclusions. Therefore, we also agree that large randomized controlled trials and related pharmacological intervention studies are essential to validate the use of TyG and its related indices in the diagnosis and treatment of psychiatric disorders.

Indeed, research on metabolic diseases (such as diabetes and fatty liver) and depression has been advancing in recent years. Our research group intends to conduct further preclinical and clinical studies to explore the relationship between insulin resistance and depressive-like behaviors, as well as the underlying molecular mechanisms. We hope to collaborate with readers and researchers in the field to clarify these issues and seek feasible solutions.
